# Supplementary material for: The interactome of intact mitochondria by cross-linking mass spectrometry provides evidence for coexisting respiratory supercomplexes
Source: Mol Cell Proteomics. 2017 Dec 8;17(2):216–32. doi: 10.1074/mcp.RA117.000470 (PMC5795388; doi:10.1074/mcp.RA117.000470)
Supplement: Supplemental Data [file supp_RA117.000470_133922_0_supp_23978_fzffwf.zip › spectra_annotation/mito_DR_spectra_annotation/368-1-7-1-7-1.pdf]

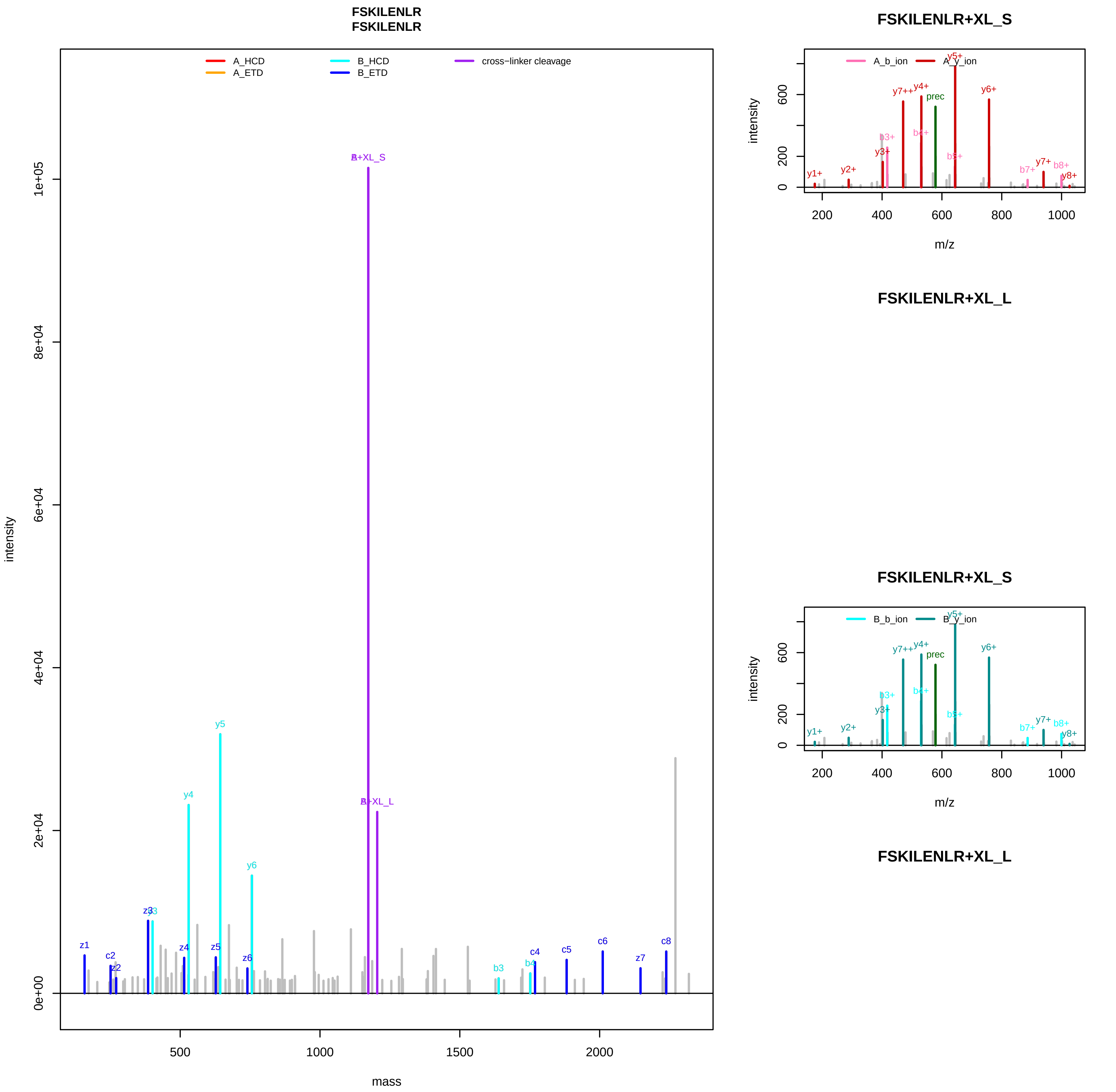
FSKILENLR
FSKILENLR
A_HCD
A_ETD
B_HCD
B_ETD
cross−linker cleavage
B+XL_S
B+XL_L
z1 c2 z2 z3 y3 y4 z4 z5 y5 z6 y6 b3 b4 c4 c5 c6 z7 c8
intensity
mass
0e+00
2e+04
4e+04
6e+04
8e+04
1e+05
500
1000
1500
2000
FSKILENLR+XL_S
A_b_ion
A_y_ion
y1+ y2+ y3+ b3+ y7++ y4+ b4+ prec y5+ b5+ y6+ b7+ y7+ b8+ y8+
intensity
m/z
FSKILENLR+XL_L
FSKILENLR+XL_S
B_b_ion
B_y_ion
y1+ y2+ y3+ b3+ y7++ y4+ b4+ prec y5+ b5+ y6+ b7+ y7+ b8+ y8+
intensity
m/z
FSKILENLR+XL_L
